# Meat hybrids–An assessment of sensorial aspects, consumer acceptance, and nutritional properties

**DOI:** 10.3389/fnut.2023.1101479

**Published:** 2023-02-07

**Authors:** Marie-Christin Baune, Keshia Broucke, Sandra Ebert, Monika Gibis, Jochen Weiss, Ulrich Enneking, Adriano Profeta, Nino Terjung, Volker Heinz

**Affiliations:** ^1^DIL German Institute of Food Technologies e.V., Quakenbrück, Germany; ^2^Technology and Food Science Unit, ILVO Flanders Research Institute for Agriculture, Fisheries and Food, Melle, Belgium; ^3^Department of Food Material Science, Institute of Food Science and Biotechnology, University of Hohenheim, Stuttgart, Germany; ^4^Faculty of Agricultural Sciences and Landscape Architecture, Osnabrück University of Applied Sciences, Osnabrück, Germany; ^5^Prokribus GmbH, Institute for Social and Data Sciences, Holzminden, Germany

**Keywords:** sensory study, QDA, consumer, hedonic analysis, meat hybrid

## Abstract

So-called meat hybrids are a new class of products where a fraction of the meat product (e.g., 20%) is replaced with alternative protein sources, such as plant-based ones. Research suggests that these products could serve as a low-threshold offer for a specific target group that wants to cut down on meat, thereby facilitating the transition toward a more healthy and sustainable diet. Nonetheless, data demonstrate that meat hybrids with a high substantial meat substitution level often fail in the market. This study summarises findings on the physicochemical properties, sensory, and acceptance of six different meat hybrids (70% meat and 30% plant proteins) that were collected in the framework of a case study in the project AiF 196 EN. For this purpose, sensory characteristics were collected *via* two QDA sessions and a hedonic consumer test. Furthermore, the hybrid recipes were analysed in their proximate composition. The respective recipes varied in protein source (soybean, pumpkin, and pea) and mode of incorporation [textured vegetable protein (TVP), high moisture extrudate (HME)]. It was shown that a meat hybrid with a relatively high share of 30% plant-based proteins with peas as a protein source and TVP as a processing method can still attract consumers.

## 1. Introduction

Concerns about global malnutrition and protein availability demand a decrease in meat consumption (1). A promising alternative pathway for a more sustainable and healthier diet is to reduce the consumption of meat proteins and increase the share of plant proteins in the diet instead (2). In this context, textured soy protein, pulses, etc., are frequently used as a substitute for animal protein in product development, particularly in food start-ups (3). Another option is to replace just a fraction of the meat product (e.g., 20–50%) with plant-based proteins (4). Literature shows that in many countries, consumers are highly attached to meat and consider it an essential and integral element of their daily diet. Many consumers like the taste of meat (5). Therefore, meat hybrids may display an alternative for the broad consumer segment that is not interested in a completely vegan, respectively, vegetarian diet (6). In this manner, the mentioned hybrids could facilitate the transition to a more healthy and sustainable diet in Baune et al. (7). The short- to mid-term time period.

Against this background, Profeta et al. (6) highlighted that consumers are unfamiliar with the meat hybrids already on the market. Furthermore, the taste expectations concerning this kind of product are relatively low (8–9). That is, whereas hybrids have a plant-based protein share, which often comes along with benefits from a sustainability perspective, many consumers perceive them as unsatisfactory from a sensory point of view. Despite technological developments that led to substantial improvements concerning the sensory quality of meat hybrids, there is still a need for optimised variants in this product category. These must prove their equivalence or, even better, their superiority compared to the reference meat concerning taste, texture, and nutritional aspects (10, 11). Integrating consumer preferences into the development process can design better and superior hybrid products.

This paper presents a case study on developing a meat hybrid from the research project “Hybrid products from animal and plant sources–MeatHybrid” (Project AiF 196 EN). It summarises findings on the physicochemical and sensory characterisation of six different hybrid recipes where meat has been partially replaced by texturised soybean, pumpkin, and pea proteins. In the first step, six meat hybrids with a plant-based protein share of 30% have been analysed *via* a quantitative descriptive sensory analysis (expert tasting). The options varied across protein sources (soybean, pumpkin, and pea) and processing [Textured Vegetable Protein (TVP), High Moisture Extrudate (HME)]. TVP can be defined as “texturates” made from plant-based protein sources and water by going through a transformation from a powder-type material to a structured material (12). In contrast, HME is a high-temperature and shear-intensive process where protein unfolding, aggregation, and cross-linking, combined with a dramatic temperature drop at the cooling die, leads to the formation of meat-like fibrous structures (13). In a second step, the solution that performed best in the sensorial expert tasting was then sensorially tested by an untrained consumer panel with a larger sample size.

Furthermore, for all products, the Nutri-Score has been calculated. In the literature, there are, to our best knowledge, no studies that analyse the nutrient profile on the base of this measure for meat hybrids. Nonetheless, there is some scientific evidence from recent studies comparing the nutritional properties of meat analogues. For example, Bohrer, (14) did not find a beneficial position for neither meat analogues nor meat products. Contrarily, Kalocsay et al. (15), who applied the “Health Star Rating System,” found a better scoring of plant-based meat substitutes compared to meat products in most categories. This is supported by a recent case study for burgers from Smetana et al. (2), who demonstrated that plant-based alternatives outperformed the animal-based burger concerning nutrient profiling based on the Nutri-Score system.

The aim of the paper is first to display the product development results for a meat hybrid based on the holistic evaluation of sensory and nutritional properties. Second, the analysis should clarify if a combination of plant-based protein sources and processing techniques can be identified to substitute a substantial share of the meat without cutting corners concerning taste and improve nutritional performance.

## 2. Consumers’ sensory evaluation of meat blends

Consumers are not willing to compromise their taste for health (16) and an inferior sensory quality represents a critical barrier to market entry of meat substitutes (17, 18). Therefore, meat blends must catch up with real meat products concerning sensory characteristics. As already outlined, recent research findings demonstrate that meat blends are associated with an inferior taste compared to meat by consumers (19). These limitations need to be overcome for a successful market launch. Likewise, Grasso and Jaworska (20), who summarised hybrid products launched in the UK market, found that particular products with a meat substitution level higher than 30% often fail in the market. It can be hypothesised that it is due to taste reasons. Conversely, Grasso et al. (21) show in a European cross-country study that a large consumer segment is open for hybrids with up to 50% plant-based ingredients.

There are several recent studies about the sensorial properties of meat products with plant-based ingredients (22–24). Different protein sources (e.g., cowpea flour, pumpkin pulp, and seed) and different fractions of the corresponding alternative protein in the product were considered. Serdaroglu et al. (24) replaced lean meat in meatballs with 2–5% of pumpkin pulp, whereas Akwetey et al. (22) substituted meat with 5–20% cowpea flour for Frankfurter-type sausages. Both studies tried to identify which replacement percentage performs best from a sensory perspective. The sensorial tests have been conducted with untrained consumers (22, 24). The products were evaluated on appearance, juiciness, colour, texture, flavour, and overall acceptance. A hedonic scale structured in 9-points was applied, and the serving sample was randomised. In the case of the cowpea flour, it was found that a replacement of 10% was equally acceptable to the panellist, like the control without cowpea flour. A more than 15% replacement leads to a lower acceptance (22). In the study of Zamuz et al. (25), panellists revealed a higher acceptance of meat products manufactured with bean and lentil protein.

As for hybrid-cooked sausages, Broucke et al. (26) showed that replacing 20% of lean pork meat with pea protein isolate did not result in any significant alterations concerning sensorial attributes. However, when using pea TVP and HME, severe sensorial flaws (e.g., cavities) were found. Neville et al. (27) reported that the absence of meaty taste results in the rejection of vegan and hybrid beef burgers and that meaty flavour, meaty colour, and moist texture increase acceptance of vegan and hybrid sausages. They compared the sensory acceptability of hybrid, meat and meat-free products with consumers. They found no significant difference between hybrid and meat products, while meat-free products were less accepted. Interestingly, Grasso et al. (28) found in a blind consumer test with commercial samples that blended burgers scored significantly higher in overall acceptability than beef- and plant-based meat-free burgers.

These findings demonstrate that it is possible to substitute meat with different plant-based proteins without a loss concerning taste. Nonetheless, it is necessary to identify the suitable substituted protein and the appropriate processing technology (e.g., HME and TVP) and determine the amount of substitution because this mainly affects the product’s acceptance. Furthermore, using plant- instead of animal-based proteins may also cause process-related changes due to variations in their techno-functionality. For example, Ebert et al. (29) have recently shown that using texturised pumpkin seed proteins alters the drying behaviour of dry-cured meat hybrids, thereby causing alterations in texture and free water. Finally, nutritional quality needs to be considered, as the bioavailability of meat and plant proteins differ.

## 3. Methods

### 3.1. Sample preparation

#### 3.1.1. Raw materials

Lean pork shoulder sections (S3 GEHA standard) were purchased from a local butcher (Landschlachterei G.H. Diekmann, Essen, Germany). TVP and HME based on water and pea protein isolate (PISANE^®^ C9, Cosucra Group, Warcoing, Belgium) were provided by the DIL process engineering department (DIL, Quakenbrück, Germany). Other ingredients were potato starch (FRUTAROM Production GmbH, Freilassing, Germany), fresh onions, canola oil, and breadcrumbs (each from Jeden Tag, ZHG-mbH, Offenburg, Germany) as well as spices.

#### 3.1.2. Meatball production

A standard meatball recipe was adapted so that 30% of meat was replaced with pea, sunflower or pumpkin TVP, or pea, sunflower, or pumpkin HME (Table 1) while maintaining the final protein, water, fat, and salt content (7). Meat and onions were minced through a 3 mm plate (PRIMUS MEW 713, MADO GmbH, Dornhan, Germany). TVP was rehydrated 1:1 in hot water (80°C) while stirring (Bär Varimixer RN10, Varimixer A/S, Brøndby, Denmark). HME and TVP were chopped to 3–4 mm in a vacuum bowl cutter (5,000 Express, 30 L, Kilia GmbH, Neumünster, Germany) using a bowl speed of 14 min-1 and a knife speed of 2,000 min-1 for 120 s followed by 3,000 min-1 for 20 s. All ingredients except potato starch and breadcrumbs were pre-mixed. Then the meat mass was mixed in the bowl cutter while slowly adding potato starch and breadcrumbs with backward running blades (100 min-1) at a bowl speed of 16 min-1 for 90 s followed by a bowl speed of 10 min-1 for 20 s. For the expert tasting, 100 g of meatballs were formed with a patty maker. For the consumer tasting, only 50 g of meatballs were formed due to the quantity required.

**TABLE 1:** Recipe for each (hybrid) meatball type [Reprinted from Baune et al. (7)].

Ingredients	Control	30% pea protein		30% sunflower protein		30% pumpkin protein	
		HME	TVP	HME	TVP	HME	TVP
S3 pork meat (%)	85.00	59.50	59.50	59.50	59.50	59.50	59.50
Textured protein (%)	–	16.37	13.88^a^	19.30	20.49^a^	16.65	16.62^a^
Fresh onions (%)	5.00	5.00	5.00	5.00	5.00	5.00	5.00
Water (%)	10.00	17.15	19.58	17.75	16.78	18.79	18.70
Canola oil	–	2.35	2.54	1.75	1.82	2.21	2.27
Total	100.00	100.37	100.50	103.30	103.59	102.15	102.09
Breadcrumbs (%)^b^	8.00	8.00	8.00	8.00	8.00	8.00	8.00
Potato starch (%)^b^	2.50	2.50	2.50	2.50	2.50	2.50	2.50
Salt (%)^b^	1.30	1.03	1.01	1.30	1.30	0.95	0.95
Pepper [black (%)]^b^	0.15	0.15	0.15	0.15	0.15	0.15	0.15
Pepper [white (%)]^b^	0.05	0.05	0.05	0.05	0.05	0.05	0.05
Garlic (%)^b^	0.03	0.03	0.03	0.03	0.03	0.03	0.03
Nutmeg (%)^b^	0.03	0.03	0.03	0.03	0.03	0.03	0.03

HME, high-moisture extrudate; TVP, textured vegetable protein.

^a^Amount of 1:1 rehydrated TVP.

^b^Higher yields (Total > 100%) were ignored when setting the amount.

After 1 h cooling at 3°C, the meatballs were first deep-fried at 175°C for 90 respective 60 s (100, 50 g of meatballs) until the desired browning was achieved and then cooked in an oven at 180°C circulating air (JOKER B 2-3, Eloma GmbH, Maisach, Germany) until the core reached 72°C. The meatballs were stored at −18°C.

### 3.2. Sensory evaluation

#### 3.2.1. Quantitative descriptive analysis (expert panel)

After the definition of meatball attributes from meatballs with 40% protein substitution in a free choice profiling session with trained panellists, two QDA sessions were performed, each including control, one hybrid meatball with 30% pea protein, and two hybrid meatballs with other proteins added (30% sunflower and 30% pumpkin). After approval by the Ethical Commission for Sensorial tests of ILVO, the QDA of the meatballs was performed in a standardised taste lab (ISO 8589:2007, 2007). The evaluation of the attributes *Colour Intensity Outside*, *Colour Intensity Inside*, *Off-Odour*, *Atypical Taste*, *Musty Taste*, *Meat Taste*, *Dry Mouthfeel*, *Crispiness*, *Granularity*, and *Chewiness* (after chewing five times) was done on a 10-points linear scale ranging from 1 to 10 (Table 4). The meatballs were reheated in an oven at 180°C circulating air until a core temperature of 72°C was reached and served at 55–60°C. The samples were placed through Latin Square design (calculated with FIZZ Software, SARL BIOSYSTEMES, Couternon, France) and tasted likewise at 55–60°C at the same time by ten (QDA session 1) respective nine panellists (QDA session 2). Moreover, the general acceptance as a commercial product (dummy coding: yes/no) was queried.

**TABLE 2:** Overview of nutri score categories and related to nutrition points for solid and liquid foods.

Nutrition points	−15 to −1	0–2	3–10	11–18	19–40
Nutri score	A	B	C	D	E

**TABLE 3:** Sensorial attribute scores (means ± SD) on a 10-point linear scale.

		QDA session 1 (*n* = 10)					QDA session 2 (*n* = 9)		
			Pea	Sunflower	Pumpkin		Pea	Sunflower	Pumpkin
Attribute	Scale (0–10)	Control	TVP	HME	TVP	Control	HME	TVP	HME
Colour outside	Pale–dark	5.63 ± 1.16	5.05 ± 1.54^a^	5.08 ± 1.04^b^	6.63 ± 1.69^c^	5.60 ± 1.27	4.48 ± 1.43^c,d^	5.19 ± 0.79^e^	7.24 ± 0.51^a,b,d,e^
Colour inside	Pale–dark	4.37 ± 1.14^a^	3.77 ± 1.14^b^	4.45 ± 1.40^c^	6.52 ± 0.88^a,b,c,d,e,f^	4.40 ± 0.94^d^	3.81 ± 1.32^e^	3.82 ± 1.05^f^	6.92 ± 0.63^a,b,c,d,e,f^
Off-odour	Absent–very intense	2.84 ± 2.10^a^	4.23 ± 1.90	4.52 ± 1.78	5.48 ± 1.63^a,b^	2.28 ± 1.37^b^	4.73 ± 1.83	4.94 ± 1.86^b^	5.42 ± 1.25^a,b^
Atypical taste	Absent–very intense	2.82 ± 1.81^a^	4.79 ± 0.97	4.63 ± 1.59	6.15 ± 2.28^a,b^	2.97 ± 1.24^b^	5.32 ± 1.43^a,b^	5.78 ± 0.52^a,b^	6.20 ± 0.75^a,b^
Musty taste	Absent–very intense	2.47 ± 1.59^a^	4.52 ± 1.32^b^	4.92 ± 1.86^a^	6.86 ± 1.36^a,b^	3.38 ± 1.55^b^	5.44 ± 1.63^a^	6.36 ± 1.06	5.29 ± 1.24^a^
Meat taste	Absent–very intense	5.08 ± 0.94^a^	3.82 ± 1.65	3.20 ± 1.96	2.54 ± 1.74^a,b^	5.03 ± 1.04^b^	2.52 ± 1.17^a,b^	2.21 ± 1.14^a,b^	2.89 ± 1.00^a,b^
Dry mouthfeel	Very dry–very juicy	4.36 ± 1.46	3.87 ± 1.65	3.74 ± 1.67	2.70 ± 1.35	4.46 ± 1.09	4.49 ± 1.35	4.14 ± 1.50	4.03 ± 2.00
Crispiness	Very mushy–very crisp	4.39 ± 1.41	4.16 ± 1.69	3.01 ± 1.54	4.14 ± 1.81	5.59 ± 1.23	3.30 ± 1.86	4.07 ± 2.34	4.70 ± 1.37
Granularity	Very granular–very smooth	4.99 ± 0.83	4.74 ± 1.62	5.41 ± 1.92	4.10 ± 1.80	5.31 ± 1.01	5.73 ± 1.12	5.86 ± 0.93	4.13 ± 1.55
Chewiness	Very soft–very hard	4.72 ± 1.06	4.78 ± 1.55	4.38 ± 1.63	4.63 ± 1.71	4.77 ± 0.58	4.14 ± 1.39	4.18 ± 0.88	5.36 ± 1.13
Acceptance (%)		100 (10/10)	80 (8/10)	60 (6/10)	10 (1/10)	100 (9/9)	44 (4/9)	44 (4/9)	56 (5/9)

Different superscript letters indicate groups of significant difference within the same attribute (confidence interval 95%). SD, standard deviation; QDA, quantitative descriptive analysis; TVP, textured vegetable protein; HME, high moisture extrudate.

**TABLE 4:** Multiple logistic regression analysis of acceptability.

Ind. variable	Coefficient	SE	Wald stat.	*P*-value
Constant	4.127	3.222	1.641	0.200
Colour outside	0.581	0.404	2.073	0.150
Colour inside	−**0.846**	0.329	6.625	0.010**
Off-odour	−0.166	0.233	0.505	0.477
Atypical taste	0.325	0.333	0.948	0.330
Musty taste	−**0.889**	0.364	5.956	0.015*
Meat taste	**0.523**	0.260	4.034	0.045*
Dry mouthfeel	−0.156	0.263	0.349	0.555
Crispiness	0.302	0.208	2.113	0.146
Granularity	−0.088	0.251	0.123	0.726
Chewiness	−0.185	0.305	0.367	0.544

Significance codes: ** ≤ 0.01, * < 0.05. *n* = 76. Pearson Chi-square Statistic: 63.501 (*p* = 0.494). Likelihood ratio test statistic: 41.211 (*p* = <0.001). −2*Log(Likelihood) = 59.843. Hosmer–lemeshow statistic: 5.689 (*p* = 0.682). No multicollinearity was found (Threshold = variance inflation factor 4.0).

Bold values indicate significant coefficients.

The panel members have 3–11 years of experience with sensorial tests, with 67% of the panel having 11 years of experience. They are familiar with consumer tests and sensorial tests on trained and expert panel level. Most of the tastings in which the panel members with longer experience participated, involved meat products.

The experts selected and defined the list of attributes during Free Choice Profiling sessions. During these sessions, groups of 4–5 experts described hybrid meats with relatively high rates of inclusion of plant proteins (so-called “extremes”). Based on their findings per group, one discussion session with all groups took place and was moderated by the conductor of the taste tests. After this discussion, a list of sensorial attributes, together with their definitions, was established.

#### 3.2.2. QDA data analysis

All results of the meatballs are expressed as means ± standard deviation. Data analysis was performed in Sigma Plot 13 (Systat Software GmbH, Erkrath, Germany). One Way Analysis of Variance (ANOVA) with the Tukey test (95% CI, *p*-value = 0.05) was used for statistical evaluation. All data showed normal distribution. For QDA results, a multiple logistic regression analysis was applied with “acceptability” as a dependent (0 = unacceptable, 1 = acceptable) and all other parameters as independent variables. The software was also used for regression as well as Pearson correlation analysis. Principal Component Analysis (PCA) of the sensory data was done in the software R (30) using the packages “stats” and “mlogit” (31).

#### 3.2.3. Untrained consumer panel

The consumer tasting took place in the time periods from the 10th to 11th of December 2019 in the FoodSense-laboratory of the University of Applied Sciences of Osnabrück.^1^ The tasting was conducted as an omnibus survey in the form of a central location test in a standardised taste lab (ISO 8589:2007, 2007). A total of 67 consumers that eat meat snacks were recruited for the study from the taster pool of the FoodSense-laboratory (female = 52%, male = 48%; 25 years and below = 39%, above 26 years = 61%). The sample size was relatively small due to budgetary reasons. Nonetheless, the computer simulations of Gacula and Rutenbeck (32) support the commonly cited sample size of 40–100 for nine-point hedonic scales as applied in our study.

The meatballs in which 30% meat protein was replaced by pea protein in the form of TVP were defrosted 1 ½ h before the study started. Afterward, they were fried in a pan with vegetable oil from each side for 4–5 min until golden brown and tasted likewise at 55–60°C simultaneously by ten consumers in one tasting session.

They had to conduct a hedonic analysis and evaluate the products on a nine-point Likert scale ranging from extraordinarily bad (1) to extraordinary good (9) concerning *Appearance, Odour, Taste*, and the overall impression of the product.

In a second step, a Just-About-Right-Scale (JAR) was applied for *Colour Outside, Colour Inside, Flavour, Meat Taste, Juiciness*, and *Bite Firmness*. For this purpose, for the scale, the terms “much too little,” “a bit too little,” “just right,” “a bit too much,” and “far too much” were used.

### 3.3. Consumer questionnaire

In addition to the sensory task, the untrained consumers had to fill out a short questionnaire. In the first step, the consumers had to answer three questions: “How often do you eat meat snacks?” [(I) more than once in 14 days, (II) approx. once a month, (III) approx. once in 3 months, (IV) less than once in 3 months, (V) never; only one answer possible], “Where do you buy your meat products?” [(I) supermarket, (II) discounter, (III) farmers market, (IV) online, (V) directly at farms, (VI) butchery store; only one answer possible], and “How often do you buy organic, free-range or regionally produced meat for your household?” [(I) more than once in 14 days, (II) approx. once a month, (III) approx. once every 3 months, (IV) less than every 3 months, (V) never; only one answer possible].

Furthermore, the tasting ended with the willingness-to-buy question, “The product you have just tasted consists of 70% animal meat and 30% plant-based protein. Can you imagine buying this product?” (scale: 1 = “I would definitely buy this product” to 5 = “I would definitely not buy this product”) and an open question (“Is there anything else you would like to tell us about the product?”).

### 3.4. Combined data analysis untrained consumer panel and consumer questionnaire

Two linear regression models were estimated to analyse the impact of the sensory JAR and the hedonic parameters on the willingness-to-buy. A linear regression line has an equation of the form *Y = a + bX*, where *X* is the explanatory variable and *Y* is the dependent variable (33). The slope of the line is b, and a is the intercept (the value of *y* when *x* = 0). In this study, the following two models were estimated:

*Formula I (impact hedonic parameters):*

*Y_*willingness–to–buy*_ = constant + b_*taste*_X_*taste*_ + b_*appearance*_X_*appearance*_ + b_*odour*_X_*odour*_*

*Formula II (impact JAR parameters):*

*Y_*willingness–to–buy*_ = constant + b_*firmness*_X_*firmness*_ + b_*juiciness*_X_*juiciness*_ + b_*odour*_X_*odour*_+ b_*colour*_X_*colour*_ + b_*flavour*_X_*flavour*_ + b_*meat taste*_X_*meat taste*_ + b_*bite firmness*_X_*bite firmness*_+ b_*freq. eating*_X_*freq. eating*_*

For the regression analysis, the JAR parameters were re-coded so that the evaluation “just right” received the value one (1), whereas all other evaluations were recorded as zero. Thus, in the regression analysis, the impact on the dependent for the JAR parameters is shown when these parameters are “just right.” All calculations were carried out in the software package R (30).

### 3.5. Nutritional profiling

#### 3.5.1. Proximate composition

The chemical composition of the TVP and HME was analysed according to the Amtliche Sammlung von Untersuchungsverfahren (ASU) methods F 0014 (EG), L 06.00-3, L 17.00-18, L 00.00-18, L 06.00-4, §64 LFGB of the German Federal Office of Consumer Protection and Food Safety (34). Compositional information on potato starch, as well as bread crumbs and rapeseed oil, was provided by Frutarom Savory Solutions and Zentrale Handelsgesellschaft mbH, respectively.

#### 3.5.2. Determination of the nutri-score

The study relied on identifying a nutritional score according to the rating system accepted and voluntarily applied for front-of-pack labelling in France since 2017 (35) and later in a few European countries, including Germany from 2020 (36). The Nutri-Score classifies foods into five categories according to nutritional quality (from category A, indicating higher nutritional quality, to category E, indicating lower nutritional quality), using Nutri-Score with the five-colour nutritional label (5-CNL) derived from the United Kingdom’s Food Standards Agency nutrient profiling system (the FSA-NPS dietary index) (37). Nutrient scoring in the current study was performed *via* the Nutri-Score system on a scale from −15 points (A) to +40 points (E) by accounting for nutrient content per 100 g, allocating positive points (0–10) for food energy, total sugars, saturated fatty acids and sodium (Table 2). Negative points (0–5) were given for fruit, vegetables, nuts, fibre, and proteins, following the methods described in the literature (37).

## 4. Results

### 4.1. Sensorial tastings

#### 4.1.1. Expert panel

The findings for the sensorial expert evaluation demonstrate that there are enormous differences between the analysed products (Table 3). This includes the parameters of meat taste, musty taste, and atypical taste. The all-meat recipe performed best for the *Meat Taste* parameter during QDA sessions 1 and 2 with scores of 5.08 and 5.03, respectively. Scores for the hybrid recipes were significantly (*p* ≤ 0.05) decreased in the order Pea TVP > Sunflower HME > Pumpkin HME > Pumpkin TVP > Pea HME > Sunflower TVP. The evaluation of the parameters *Atypical* and *Musty Taste* revealed similar results, with a Pea TVP having the lowest and Pumpkin TVP and HME having the highest deviation from the control. Furthermore, the *Inside* and *Outside Colour* of the pumpkin variants were perceived as significantly darker due to their strong inherent colour (not shown). All other parameters (*Dry Mouthfeel*, *Crispiness, Granularity*, and *Chewine*ss) displayed non-significant differences (*p* > 0.05), indicating that product texture was not majorly affected by the origin nor by the composition and type of the extrudate.

**FIGURE 1:**
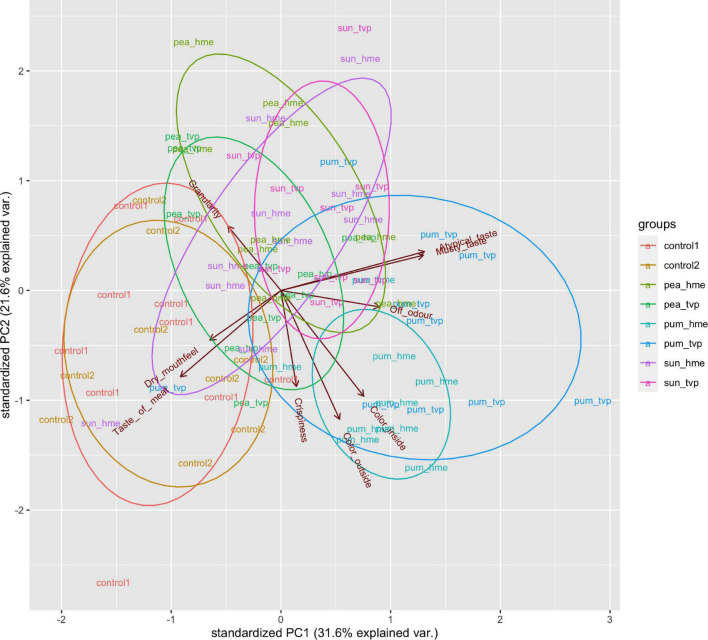
Principal component analysis of the sensorial results. pea_hme, Pea HME; pea_tvp, Pea TVP; pum_hme, Pumpkin HME; pum_tvp, Pumpkin TVP; sun_hme, Sunflower HME; sun_tvp, Sunflower TVP.

For a better overview and to condense the data on two dimensions, a PCA was carried out that explained more than 50% of the variance (Figure 1). Results showed that the parameters of *Atypical* and *Musty Taste* are opposite to the evaluation of *Meat Taste* on the first dimension, which fits the results outlined in Table 3. Furthermore, the parameter *Granularity* opposes the parameters *Crispiness*, *Chewiness* and *Inside* and *Outside Colour* on the second dimension of the PCA.

As expected, the evaluation of both controls is nearest to the parameter *Meat Taste*, whereas the hybrids with 30% pumpkin TVP were the most distant products. Figure 1 shows that the variant 30% Pea TVP was closest to the meat references compared to all other tested alternatives. *Off-odour* of Pea TVP was reduced compared to HME, which became clearer by looking at the third and fourth dimensions (Supplementary Figure 1), underlining findings on the off-flavour reduction of dry extrusion. Furthermore, it was recently shown that hybrid chicken nuggets containing Pea HME performed better than Pumpkin HME/TVP from this supplier. In contrast, those from Styrian pumpkin protein were shown to outstand both pea and pumpkin hybrids in texture (38).

Although the 30% Pea TVP hybrid performed most similarly to the control, it is to highlight that this product lies much more toward *Atypical Taste* and *Musty Taste* on the first dimension of the PCA in comparison to the meat reference. Even with the expert panel’s small sample size, the control products’ differences are significantly different.

Multiple logistic regression analysis revealed that product acceptability depended on the *Inside Colour*, *Musty Taste*, and *Meat Taste* (Table 4). These findings confirm Neville et al. (27). They reported that the absence of a meaty taste results in the rejection of vegan and hybrid beef burgers and that meaty flavour, meaty colour, and moist texture increase acceptance of vegan and hybrid sausages.

#### 4.1.2. Sensorial results TVP 30% pea–untrained consumer panel

The expert panel revealed that the hybrid recipe containing 30% Pea TVP performed best across all tested options. This option was perceived as nearest to the meat reference and got an acceptance rate of 80%. Therefore, this variant was chosen for a sensory evaluation with a larger sample of untrained consumers. Here, *Appearance*, *Odour*, *Taste*, and *Overall Acceptance* were evaluated based on a 9-point hedonic scale test and compared to an all-meat recipe.

**FIGURE 2:**
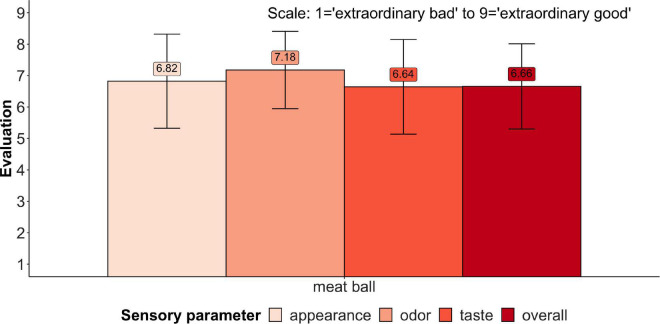
Sensorial results - hedonic analysis.

The findings for the hedonic scales used for the consumer tasting revealed relatively good results for the *Overall Acceptance* (6.66) as well as for all other parameters (e.g., *Taste* 6.64). In a sensory study with comparable products (burgers) and the same applied scale, the overall acceptability of the tested beef burger was 6.32, whereas the taste parameter scored 6.48 (2). In the mentioned study, the Beyond meat burger scored 5.36 for the overall acceptability and 5.54 for the taste parameter. Against this background, consumers’ sensory evaluation of the TVP 30% Pea is relatively good. The highest mean score received the parameter *Odour*, with a value of 7.18 (see Figure 2).

**FIGURE 3:**
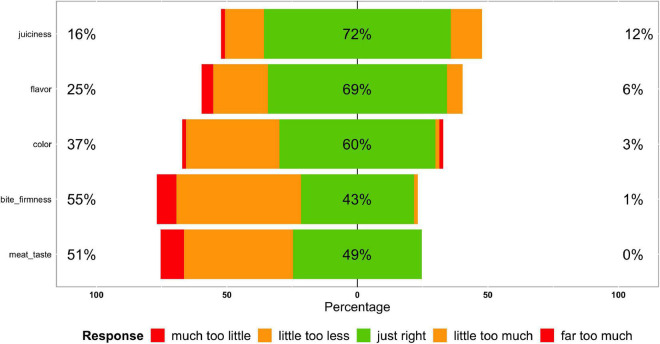
Sensorial results - JAR.

On the contrary, the outcome of the JAR-task demonstrated that *Bite Firmness* and *Meat taste* could be improved (Figure 3). Less than half of the consumers found these attributes “just right”; furthermore, a high share of consumers indicated that both parameters, *Bite Firmness* and *Meat Taste*, were too little pronounced (see Figure 3). Compared with Smetana (2), the tested TVP pea 30% achieved a similar *Bite Firmness* as the Beyond Meat burger and even a better JAR evaluation concerning the *Meat Taste* (TVP pea 30% = 49% JAR vs. Beyond Meat = 33% JAR).

Nonetheless, as already outlined, Neville et al. (27) reported that the absence of a meaty taste leads to rejection and that a moist texture increases acceptance of vegan and hybrid sausages. To achieve a more meat-like taste and increase consumer acceptance, an additional flavouring could be applied to the tested TVP meatball (39–41). There are already suppliers in the market which offer flavouring options for plant-based meat alternatives that could be tested for giving meat hybrid based on TVP a more intensive, respectively, just-right *Meat Taste* (e.g., (42)). Alternatively, Flores and Piornos (43) showed that fermentation of meat-free sausages before the extrusion process could improve the products’ aroma by either reducing/eliminating off-aromas.

Concerning the outcome of the *Bite firmness*, it is mentioned that plant-based proteins from rehydrated TVP made of pea protein are often lacking binding function. To optimise the *Bite firmness* and improve texture, higher amounts of binders (e.g., bread crumbs, starch, fibres, and methylcellulose) could be applied/tested to toughen the products.

Grasso et al. (21) pointed out that key texture features that consumers disliked about meat alternatives referred to expression “hardness” and least with “softness.” From this, it could be concluded that a high share of not “just right” concerning the JAR-parameter *Bite Firmness* does not display a big problem. We will come back to this issue when analysing the effect of *Bite Firmness* on the willingness-to-buy of the tasted product.

##### 4.1.2.1. Willingness-to-buy

**FIGURE 4:**
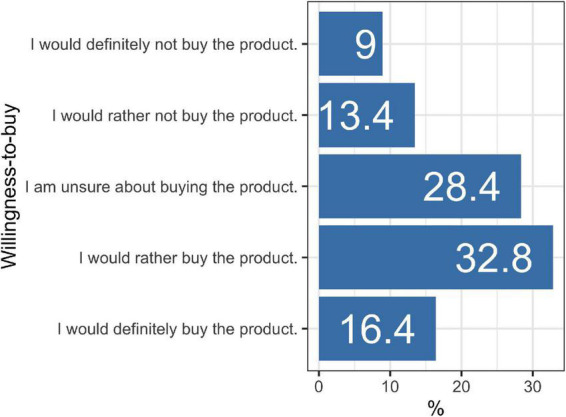
Willingness-to-buy.

After tasting the product, about one-sixth of the consumers indicated definitely to buy it, and about one-third stated that they would rather buy it (Figure 4). Thus, about 50% of the consumer sample evaluated the tested hybrid relatively positively concerning the buying aspect. This value is near to the values found by Grasso et al. (21) for UK, Spain, and Denmark. It is to highlight that in their study, no tasting was integrated. Therefore, it can be hypothesised that the taste experience has no negative impact on the willingness-to-buy meat hybrids.

Profeta et al. (6) and Grasso et al. (28) showed *via* two online surveys that if consumers have to choose between a meat and a blended meat product, the majority would opt for the pure meat variant. Against this background, the relatively high willingness-to-buy found in this study supports the assumption that the tasting experience for the tested meat hybrid exerts at least no negative effect. Nonetheless, this assumption must be tested for Germany in an additional study.

Furthermore, it is to point out that we did not indicate a price for the meat hybrid when asking for the willingness-to-buy. Profeta et al. (4) demonstrated that only 20% of the consumers would pay more for a meat hybrid compared to a corresponding meat product, and even 37% would be willing to pay a higher price for the pure meat option instead. Therefore, future studies must account for concrete product prices when recording the willingness-to-buy.

##### 4.1.2.2. Regression analysis–impact of sensorial parameters on willingness-to-buy

**FIGURE 5:**
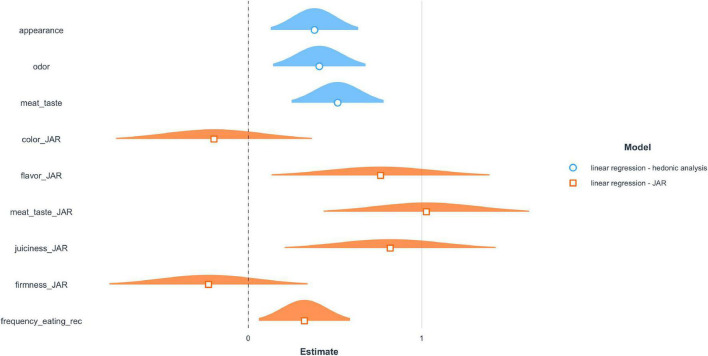
Regression results.

Both carried out regression analyses demonstrate that, in particular, the sensory evaluation of the *Taste*, respectively *Meat Taste* have a significant impact on the willingness-to-buy the hybrid meatballs. In contrast, parameters such as, e.g., *Odour, Appearance* and *Bite Firmness* play only a minor role (Figure 5 and Supplementary Table 1). Interestingly, the characteristics of *Flavour* and *Juiciness* are nearly as important as the *Taste* evaluation for the willingness-to-buy. It is to highlight that the frequency of eating meatballs positively impacts the dependent variable. Under the assumption that a higher eating frequency of meatballs goes along with a higher meat consumption and higher meat attachment, one would expect a different outcome from the literature. Profeta et al. (44) showed that the more consumers are attached to meat, the higher the probability of not choosing a meat blend option. Frequent eaters of packaged meatballs may have been confronted more often with vegan or vegetarian meatball alternatives in the supermarket and thus are more familiar with existing alternatives. Nonetheless, this assumption must be tested in future studies.

The relatively low and non-significant effect of the JAR-parameter *Bite Firmness* confirms the assumption that this parameter does not display a major problem for the consumers despite the fact that about 50% indicated a not “just right” evaluation.

#### 4.1.3. Nutri-score-evaluation

Hybrid recipes were analysed in their proximate composition. Results were then used to calculate the Nutri Score *via* the overall nutrition points (Table 5). Compared to both the control meatballs and commercially purchasable meatballs, both hybrid meatball variants showed improved Nutri-Scores (Table 4). Two commercially available products were included for comparison. Replacing meat in hybrid meatballs reduced the overall fat and thus calorie content, while the amount of fibre and vegetables/nuts was increased. This resulted in better Nutri-Scores for all hybrid recipes.

**TABLE 5:** Calculated nutri-scores of commercial and self-made meatballs.

Product	Energy (kj/100 g)	Total fat (g/100 g)	Saturated fatty acids (g/100 g)	Sugars (g/100 g)	Proteins (g/100 g)	Salt (g/100 g)	Fibres (g/100 g)	Fruits, vegetables, pulses, nuts, and rapeseed, walnut, and olive oils (g/100 g)	Sodium (mg/100 g)	Score	Nutri-score
“Frikadellen“ (Jeden Tag)	1,192.00	22.00	8.70	1.50	14.00	1.70	0.00	0.00	680.0	18	D
“G’ackie Gehaktbal” (Mora)	1,321.00	27.00	9.30	1.60	14.00	2.10	0.00	0.00	840.00	21	E
Control meatball	729.90	9.23	3.18	0.53	15.39	1.41	0.10	0.00	565.0	11	D
Pea-TVP meatball	722.74	9.03	2.46	0.54	15.37	1.37	0.29	14.63	547.0	5	C
Pea-HME meatball	723.69	9.05	2.49	0.58	15.38	1.37	0.28	16.69	547.0	5	C
Sun-TVP meatball	730.80	8.81	2.39	1.12	14.92	1.33	1.76	19.29	532.0	3	C
Sun-HME meatball	729.40	8.84	2.40	1.02	14.95	1.33	1.49	18.25	532.0	3	C
Pumpkin-TVP meatball	721.80	8.93	2.46	0.68	15.16	1.35	0.98	16.56	540.0	3	C
Pumpkin-HME meatball	721.30	8.93	2.48	0.67	15.15	1.35	1.01	16.60	540.0	3	C

## 5. Conclusion

The market success of meat substitutes, based on different alternative protein sources, calls for the holistic assessment of emerging food products from multiple sensory and nutritional perspectives to define potential benefits and drawbacks they bring to the dietary shifts. The study aimed to fill the gap in holistic experimental studies, which would simultaneously determine meat substitutes’ sensory and nutritional aspects on the same processing and production level.

This study shows that a meat hybrid with a relatively high share of 30% plant-based proteins can still attract consumers. The experts evaluated this variant nearest the control product (100% meat). The consumers confirmed the expert evaluation, and about 50% of the untrained participants indicated a relatively high willingness-to-buy the selected meat hybrid variant. As a protein source, the pea is the best option in combination with TVP as a processing method.

It is to highlight that from a nutritional perspective, based on the Nutri-Score, the selected meat hybrid variant received a better score (C) compared to the meat control product (D) and comparable meat ball products (D and E) in the market. Thus, the goal was fulfilled to develop a hybrid with improved nutritional characteristics compared to the reference meat.

Nonetheless, the studies demonstrate that about 50% of consumers still would (rather) not buy the tasted product. This gap must be closed, and the sensorial findings provide some insights toward possible solutions and approaches. The outcome of the QDA and the consumer sensory test demonstrated that there is still space for sensorial improvements to convince a higher share of consumers. On the one hand, the product selected for the consumer test (Pea TVP) performed relatively well compared to other studies that reported product developments for hybrids. In contrast, on the other hand, the findings from the QDA and consumer test show that, in particular, the meaty taste of the hybrid alternative could be improved. The selected option Pea TVP was nearest to the control products concerning the parameter *Meat Taste* in the QDA, but it appears that there is still a substantial gap.

Furthermore, in new product developments based on Pea TVP, the *Musty Taste* must be further reduced to increase consumer acceptance. Therefore, future studies could assess volatiles quantitatively in texturised plant proteins in connection with their effect on taste. This might also include an evaluation of temperature-induced protein degradation that may induce the release of peptides or affect protein functionality. Moreover, further research should be undertaken to remove unfavourable odour-active compounds or to increase the oxidation stability of plant proteins before their application in foodstuffs to obtain products with reproducible and acceptable organoleptic properties.

## Ethics statement

The studies involving human participants were reviewed and approved by the Ethical Commission for Sensorial tests of ILVO. The patients/participants provided their written informed consent to participate in this study.

## Author contributions

NT, JW, MG, and VH: funding, conceptualisation, and research method. KB, SE, M-CB, and AP: data collection, writing the original manuscript, and data analysis. UE: data and writing the original manuscript. All authors contributed to the article and approved the submitted version.

## Footnotes

1
https://www.stb-hsos.de/foodsense/
