# Supplementary material for: Meat hybrids–An assessment of sensorial aspects, consumer acceptance, and nutritional properties
Source: Front Nutr. 2023 Feb 7;10:1101479. doi: 10.3389/fnut.2023.1101479 (PMC9941143; doi:10.3389/fnut.2023.1101479)
Supplement: Supplementary file 1 [file Data_Sheet_1.docx]

Supplementary Material

**Supplementary Table 1.**

|  | *Dependent variable:* | |
| --- | --- | --- |
|  | Willingess-to-buy | |
|  | (1) | (2) |
| hedonic JAR | | |
| appearance_hedonic | 0.166^**^ |  |
|  | (0.073) |  |
| odor_hedonic | 0.162^**^ |  |
|  | (0.076) |  |
| taste_hedonic | 1.088^***^ |  |
|  | (0.079) |  |
| color_JAR |  | -0.198 |
|  |  | (0.282) |
| flavor_JAR |  | 0.762^**^ |
|  |  | (0.314) |
| meat_taste_JAR |  | 1.026^***^ |
|  |  | (0.296) |
| juiciness_JAR |  | 0.818^***^ |
|  |  | (0.304) |
| firmness_JAR |  | -0.229 |
|  |  | (0.285) |
| frequency_eating_rec |  | 0.316^**^ |
|  |  | (0.128) |
| Constant | 6.657^***^ | 4.061^***^ |
|  | (0.066) | (0.575) |
| Observations | 67 | 67 |
| R^2^ | 0.846 | 0.451 |
| Adjusted R^2^ | 0.839 | 0.396 |
| Residual Std. Error | 0.544 (df = 63) | 1.053 (df = 60) |
| F Statistic | 115.316^***^ (df = 3; 63) | 8.205^***^ (df = 6; 60) |
| *Note:* | ^*^p<0.1; ^**^p<0.05; ^***^p<0.01 | |

**Supplementary Figure 1.** Principal component analysis of the sensorial results.

**
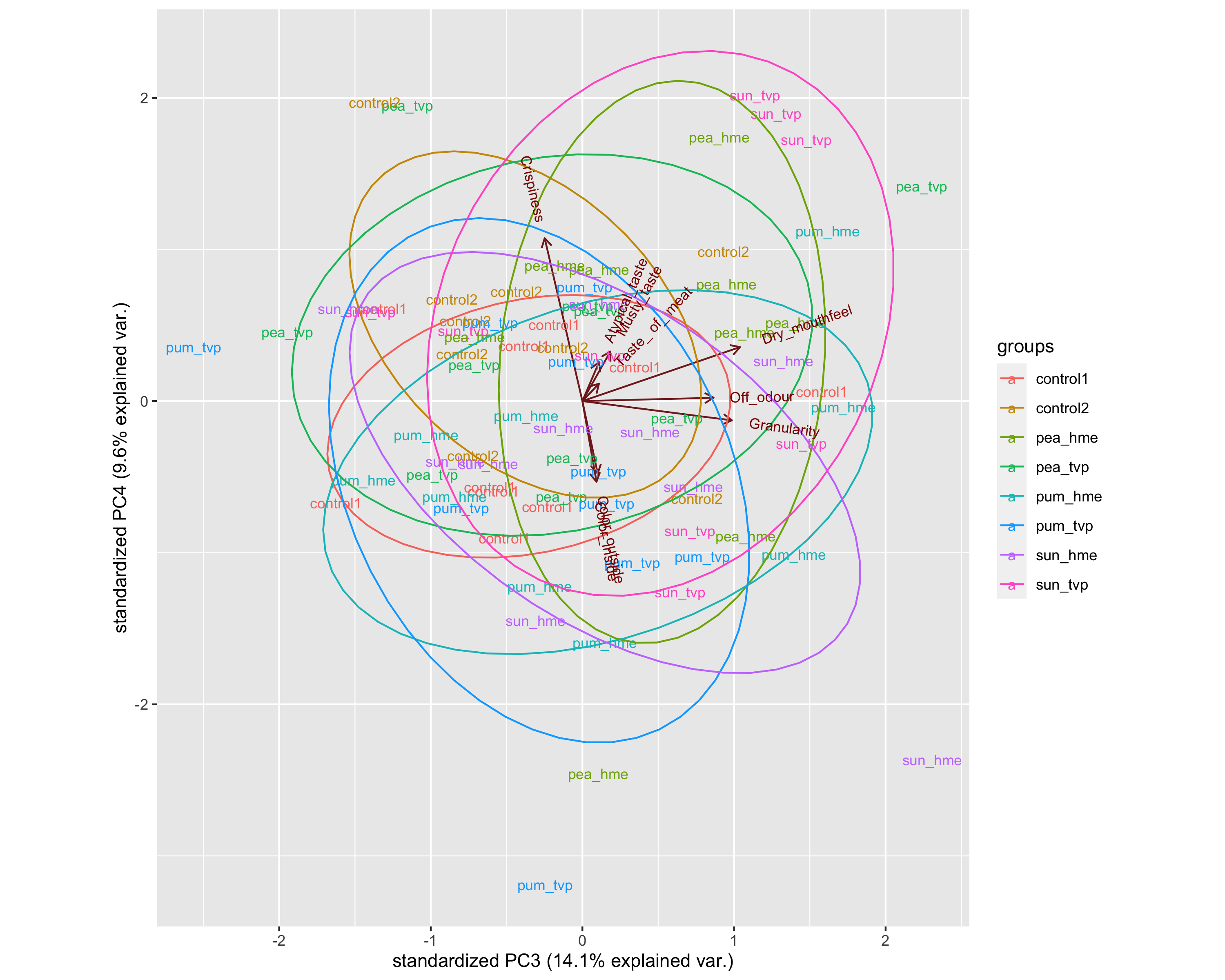
**
